# Unilateral Hearing Loss as the Sole Presentation of Extensive Intracranial Epidermoid Cyst: A Case Report

**DOI:** 10.7759/cureus.84721

**Published:** 2025-05-24

**Authors:** Abdulrahman Alosaimi, Badr E Hafiz, Ibrahim A Tawfiq, Naeem Makhdoom, Talal H Almoghthwey

**Affiliations:** 1 Otolaryngology, Head and Neck Surgery, Ohud Hospital, Medinah, SAU; 2 Neurological Surgery, King Faisal Specialist Hospital and Research Centre, Jeddah, SAU; 3 Medical Intern, Taibah University, Medinah, SAU; 4 Otolaryngology, Head and Neck Surgery, Taibah University, Medinah, SAU; 5 Neuroradiology, Ohud Hospital, Medinah, SAU

**Keywords:** cerebellopontine angle, cranial nerve compression, craniotomy, epidermoid cyst, hearing loss, inclusion cyst, intracranial tumor

## Abstract

Epidermoid cysts are rare congenital tumors of the central nervous system. These histologically benign, slow-growing lesions form when ectodermal cells become trapped during the closure of the neural tube. Histologically, they consist of a core composed of keratin, desquamated epithelial cells, and cholesterol, surrounded by a layer of stratified squamous epithelium. Clinical features depend on the lesion’s location. In the cerebellopontine angle (CPA), they typically present with tinnitus, vertigo, hearing loss, and facial weakness, with or without cerebellar signs and symptoms. Unilateral hearing loss as the sole presenting symptom is uncommon in the setting of a large, extensive cyst and may delay diagnosis.

A 35-year-old male presented with progressive left-sided hearing loss for one year, without vertigo, tinnitus, or other neurological symptoms. Audiological testing revealed severe-to-profound sensorineural hearing loss in the left ear. Temporal bone computed tomography and brain magnetic resonance imaging showed a large, extra-axial cystic lesion in the left CPA with characteristic diffusion-weighted imaging restriction, consistent with an epidermoid cyst. The lesion caused significant mass effect, including compression of the brainstem, cranial nerves, basilar artery, left vertebral artery, and left posterior cerebral artery. The patient underwent successful surgical excision via a retrosigmoid suboccipital craniotomy. Histopathological examination confirmed the diagnosis of an epidermoid cyst.

This case report highlights an unusual presentation of an extensive epidermoid cyst in the left CPA that manifested solely as unilateral hearing loss, underscoring the diagnostic challenges posed by this rare lesion. The findings emphasize the importance of considering atypical presentations of intracranial tumors in the differential diagnosis of patients with unexplained hearing loss.

## Introduction

Epidermoid cysts are rare, benign, slow-growing lesions of the central nervous system that originate during early embryonic development [1]. These cysts form when ectodermal cells become trapped during the closure of the neural tube, typically between the third and fifth weeks of gestation [1]. Histologically, they consist of a core composed of keratin, desquamated epithelial cells, and cholesterol, surrounded by a layer of stratified squamous epithelium [1,2]. They account for approximately 0.8% to 1.2% of all intracranial tumors, with about 40% located in the cerebellopontine angle (CPA), where they are the third most common lesion after vestibular schwannomas and meningiomas [1]. Less commonly, these cysts may occur in other regions, such as the parasellar area, optic chiasm, brainstem, petrous apex, and ventricular system [1]. Epidermoid cysts tend to expand along anatomical pathways offering the least resistance, including natural fissures and canals [2]. They may cross multiple cranial fossae and encase both vascular and neural structures [2].

Symptoms typically develop gradually due to the slow-growing nature of these cysts and most often appear between the ages of 20 and 50 [2]. Clinical manifestations often include chronic hearing loss and tinnitus [3]. Less frequently, patients may experience trigeminal neuralgia, hemifacial spasm, facial weakness, hydrocephalus, headaches, or signs of chemical meningitis [3]. In rare cases, rupture of the cyst may release keratinous material into the subarachnoid space, irritating nearby nerves and meninges and resulting in chemical meningitis [3]. Symptomatology varies according to the tumor’s location. CPA lesions typically present early with non-specific symptoms such as tinnitus, headaches, or facial sensory disturbances, with or without facial weakness, whereas parasellar epidermoid cysts may present with visual disturbances or seizures, especially if the lesion extends into the suprasellar region [4].

Magnetic resonance imaging (MRI) is the primary diagnostic tool. As epidermoid cysts do not respond to chemotherapy or radiation, surgery remains the mainstay of treatment. The surgical strategy depends on the tumor’s location, size, and impact on surrounding structures [4]. For CPA tumors, a retrosigmoid craniotomy is commonly employed. If the tumor extends above the tentorium, a subtemporal approach may be necessary; for lesions involving both infratentorial and supratentorial regions, a combined approach may be used [4]. The surgical objective is total excision of the cyst while preserving adjacent nerves and vessels. However, when the cyst capsule is tightly adherent to critical structures, complete removal may not be possible. Some surgeons advocate for aggressive resection to prevent recurrence, while others prefer a more conservative approach to minimize surgical risks. Given the cysts’ slow growth, long-term follow-up is essential, typically extending over 4-5 years or more to monitor for recurrence [4,5].

## Case presentation

**Figure 1:**
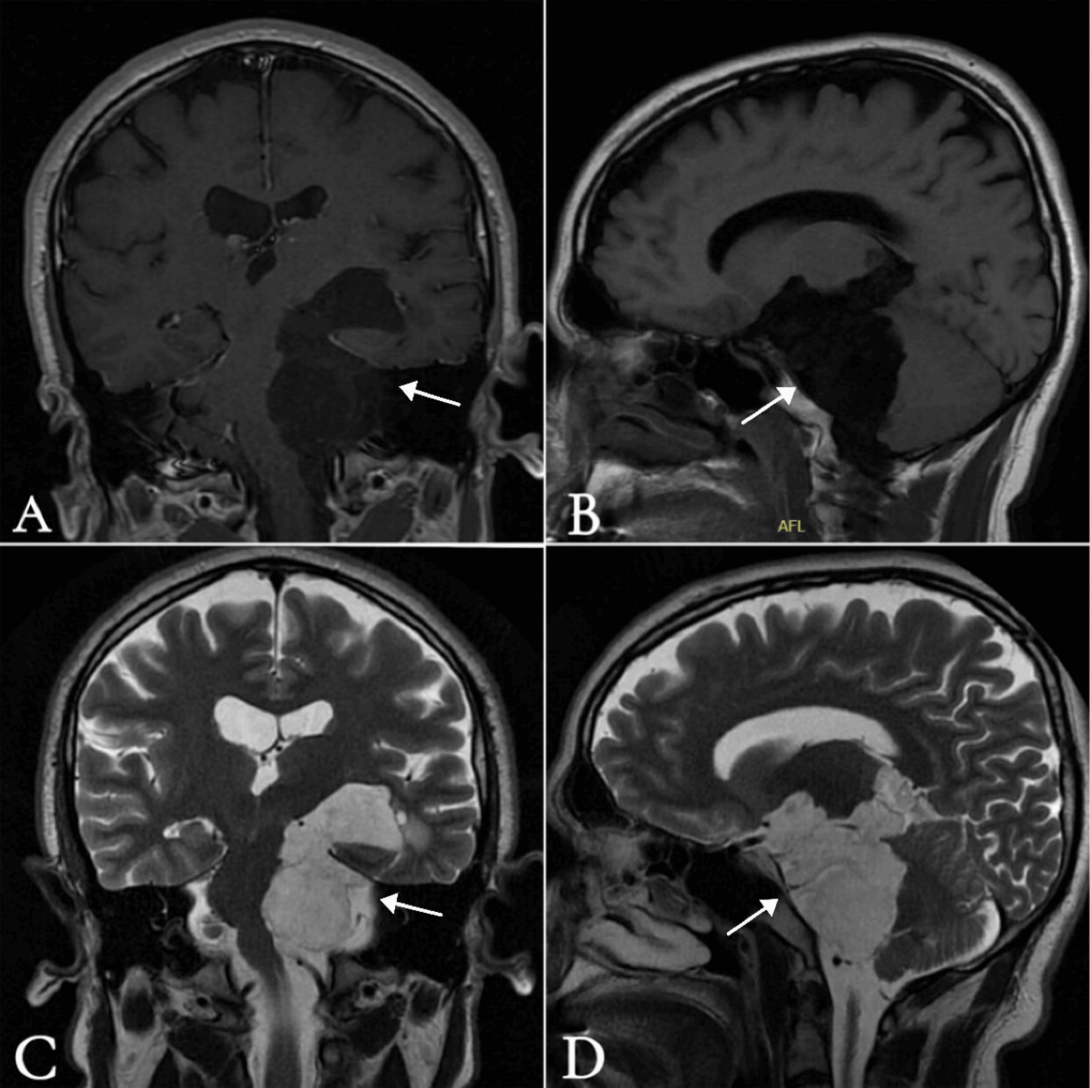
Preoperative MRI brain A) T1-weighted imaging (T1WI) with contrast, coronal view; B) T1WI without contrast, sagittal view; C) T2-weighted imaging (T2WI) without contrast, coronal view; D) T2WI without contrast, sagittal view showing a large extra-axial lesion centered within the left cerebellopontine angle (CPA), extending inferiorly into the left cerebellar medullary cistern and superiorly into the left ambient cistern. It measures approximately 4.2 × 3.2 × 6.5 cm (anteroposterior × transverse × craniocaudal). The lesion indents multiple adjacent structures, including the medial aspect of the left thalamus, left suprasellar region, interpeduncular cistern, left mesial temporal lobe, optic chiasm, left optic nerve anteriorly, left cerebellar hemisphere, brainstem (midbrain and pons) with mild medullary mass effect, left cerebellar peduncle, and hypothalamus. The mass appears hyperintense on T2WI, hypointense on T1WI, and shows no contrast enhancement.

**Figure 2:**
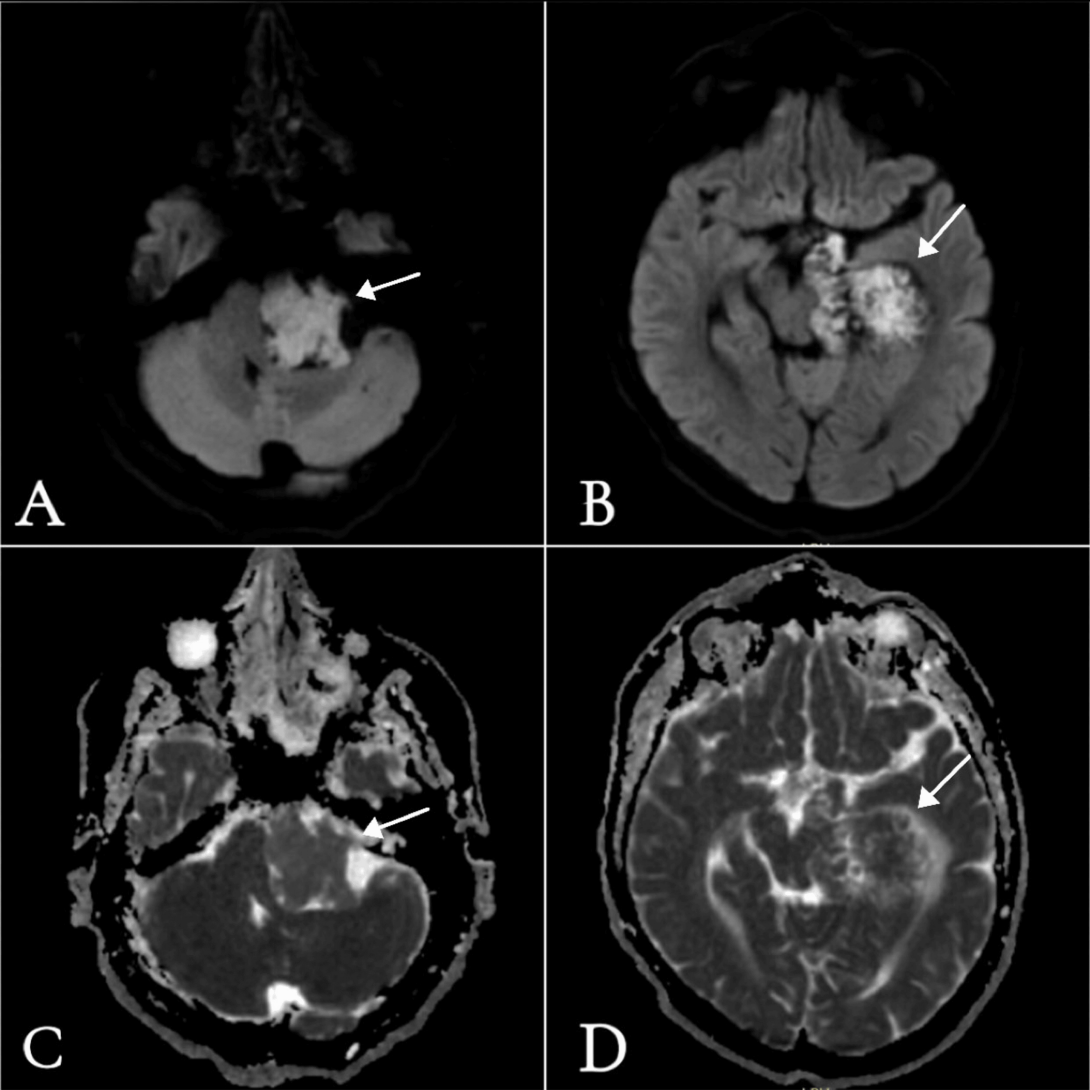
Preoperative MRI brain A-B) Diffusion-weighted imaging (DWI), axial views: A) at the level of the cerebellopontine angle (CPA) and internal auditory canal; B) at the level of the midbrain and parahippocampal gyrus, both showing high signal intensity. C-D) Apparent diffusion coefficient (ADC), axial views: C) at the level of the CPA and internal auditory canal; D) at the level of the midbrain and parahippocampal gyrus, both showing signals similar to adjacent brain parenchyma with multifocal areas of restricted diffusion.

**Figure 3:**
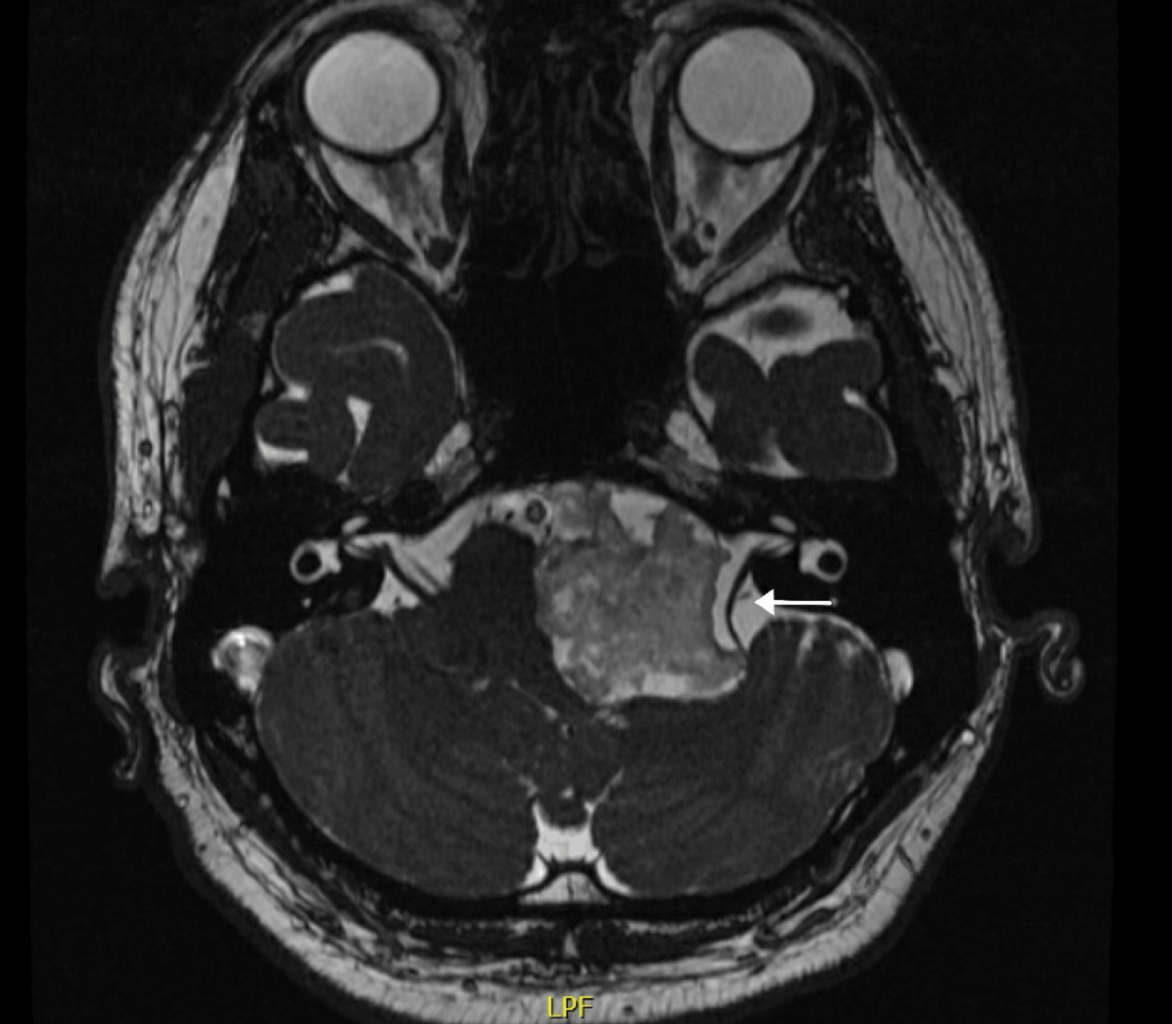
Preoperative MRI brain Fast Imaging Employing Steady-state Acquisition (FIESTA) protocol, axial view at the level of the cerebellopontine angle (CPA) and internal auditory canal, showing a large extra-axial isointense lesion causing significant kinking and displacement of the left vestibulocochlear and facial nerves, along with compression and displacement of the pons.

A 35-year-old male with no significant medical or surgical history was referred to our clinic with a one-year history of progressive left-sided hearing loss, accompanied by intermittent pressure-like headaches not associated with nausea or vomiting. He denied any history of vertigo, tinnitus, imbalance, diplopia, or blurry vision, and his remaining ear, nose, and throat (ENT) history was unremarkable. On examination, the patient demonstrated severe-to-profound sensorineural hearing loss in the left ear, with normal hearing in the right ear. Right horizontal nystagmus was observed; however, the remainder of the ENT and neurological examination was unremarkable, including cranial nerve and cerebellar assessments. MRI of the brain (Figures 1-3) revealed a large extra-axial lesion centered in the left cerebellopontine angle (CPA), extending inferiorly into the left cerebellar medullary cistern and superiorly into the left ambient cistern.

The lesion indented multiple adjacent structures as shown in Figures 1 and 3. There was a significant mass effect, including rightward displacement of the brainstem and vascular structures. The basilar artery, left vertebral artery, and left posterior cerebral artery were encased and mildly displaced. The left middle cerebral artery (MCA) (M1 segment) was partially involved. The left vestibulocochlear and facial nerves were significantly displaced and kinked. The left trigeminal nerve was displaced superiorly and medially. The lesion demonstrates high signal intensity on T2-weighted imaging and low signal on T1-weighted imaging. There was no contrast enhancement after intravenous contrast administration, and it showed diffusion restriction on diffusion-weighted imaging (DWI)/apparent diffusion coefficient (ADC), which was highly suggestive of the imaging characteristics of an epidermoid cyst (Table 1).

**Table 1:** Differential diagnosis of CPA lesions based on imaging characteristics. DWI: diffusion-weighted imaging; CPA: cerebellopontine angle

Lesion Type	T1 Signal	T2 Signal	DWI	Enhancement
Vestibular Schwannoma	Hypointense/Isointense	Hyperintense	Restricted	Heterogeneous, intense
Meningioma	Isointense/Hyperintense	Isointense/Hyperintense	Non-restricted	Homogeneous, vividly enhancing
Epidermoid Cyst	Hypointense	Hyperintense	Restricted	± Scattered peripheral enhancement
Arachnoid Cyst	Hypointense	Hyperintense	Non-restricted	Non-enhancing
Dermoid Cyst	Hyperintense	Hypo-/Hyperintense	Non-restricted	Non-enhancing; may show extensive pial enhancement if ruptured (chemical meningitis)

The condition and surgical management were explained to the patient. He agreed to proceed, and all of his questions and concerns were addressed. The patient underwent a left retrosigmoid suboccipital craniotomy and resection of the left epidermoid cyst from the left CPA, with excision through the supratentorial subtemporal region via transtentorial access. Intraoperative neuromonitoring (IONM) was used. Intraoperatively, the tumor appeared as a typical epidermoid, with a white, pearly appearance occupying the CPA, cerebellomedullary angle, and prepontine area, extending through the tentorial incisura into the medial temporal region and pineal cistern.

The tumor was dissected circumferentially and from the brainstem without any obvious complications or aggressive maneuvers, extending to the premedullary, prepontine, and perimesencephalic areas. During tumor removal, the facial, glossopharyngeal, spinal accessory, hypoglossal, abducent, trigeminal, and trochlear cranial nerves were identified in sequence. The arachnoid covering the cranial nerves was preserved, with passive IONM signals maintained, and hemostasis was achieved.

Tumor removal was performed with surplus irrigation to avoid spillage of the cheesy material outside the surgical field, preventing chemical meningitis. Resection continued with different microscope angles using microhooks and micro-dissectors. After completing the infratentorial portion, the tentorium was coagulated and cut to access the subtemporal area, and resection continued until all visible tumor was removed. The surgery was uneventful, with no intraoperative complications. Histopathological analysis confirmed the diagnosis of an epidermoid cyst.

**Figure 4:**
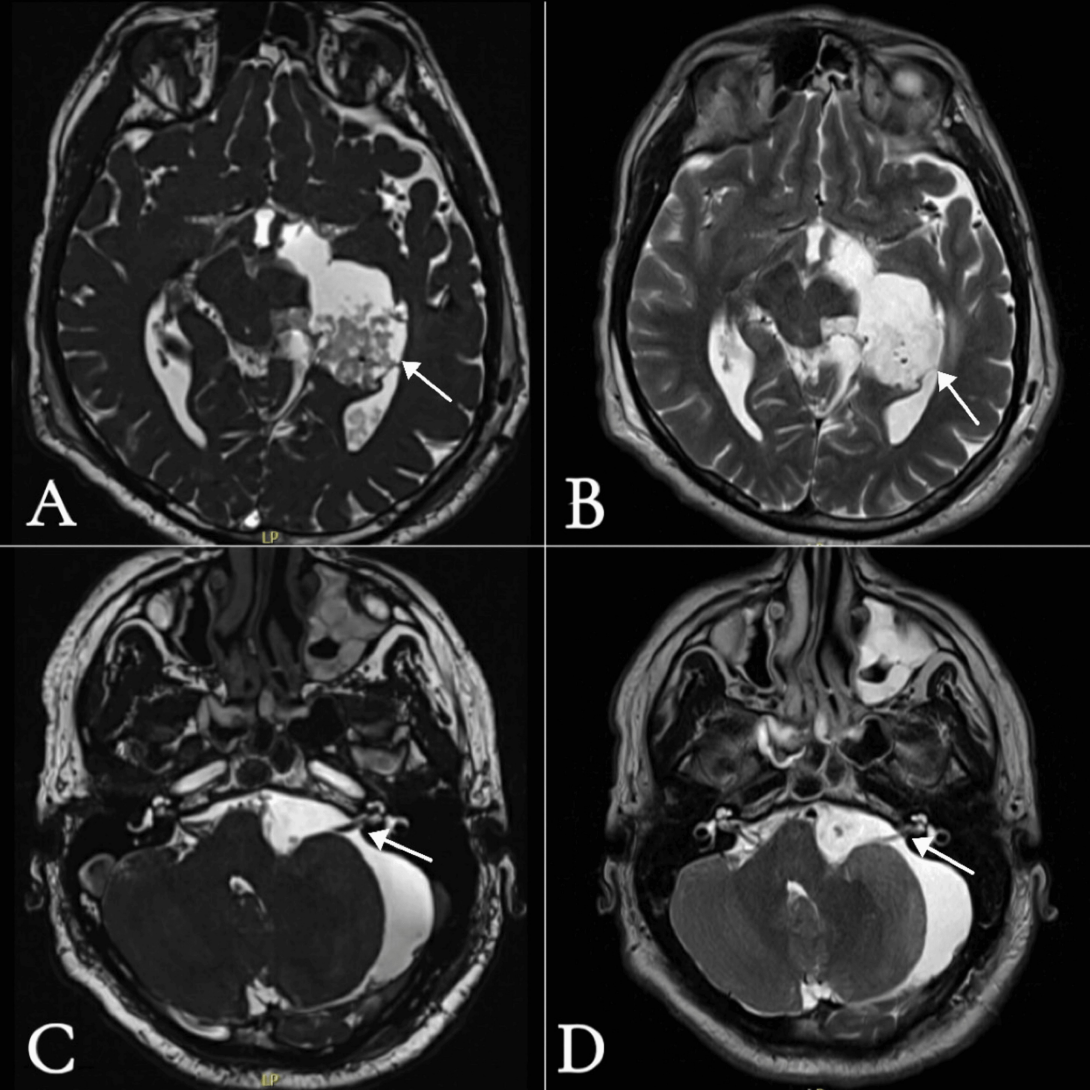
Postoperative MRI of the brain A-B) Axial views at the level of the midbrain: A) FIESTA protocol and B) T2WI without contrast, showing residual resected epidermoid cyst indenting the left occipital horn. C-D) Axial views at the level of the CPA and internal auditory canal: C) FIESTA protocol and D) T2WI without contrast, showing improvement in mass effect previously exerted by the epidermoid cyst, with resolution of the kinking and displacement of the left vestibulocochlear and facial nerves. FIESTA: Fast Imaging Employing Steady-state Acquisition: CPA: cerebellopontine angle; T2WI: T2-weighted imaging

**Figure 5:**
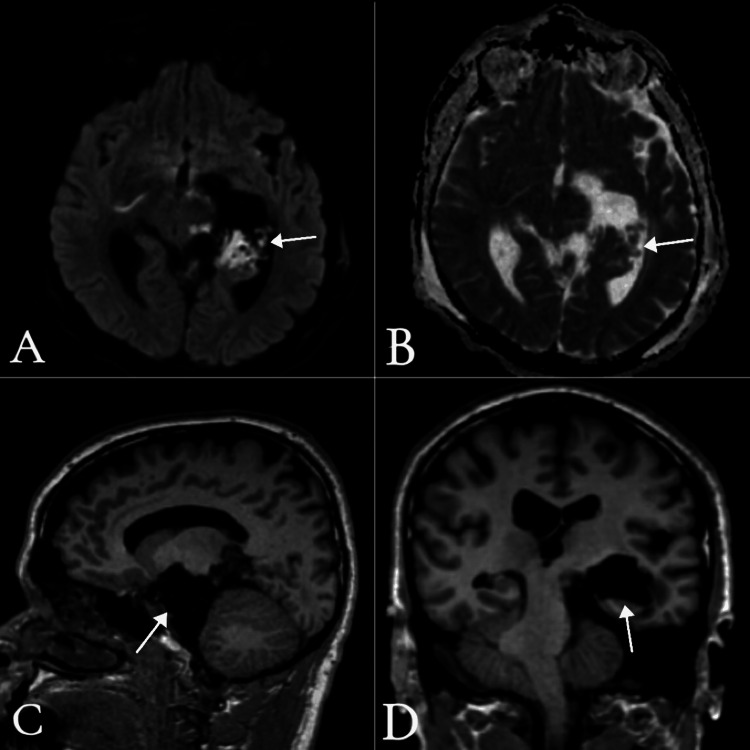
Postoperative MRI of the brain A) DWI axial view at the level of the midbrain, showing residual epidermoid evident by high signal intensity. B) ADC axial view at the same level, showing corresponding low signal intensity, confirming restricted diffusion. C) T1WI without contrast, sagittal view; D) T1WI without contrast, coronal view - both showing improvement in compression and displacement of the brainstem and left cerebellar hemisphere compared with preoperative images. DWI: diffusion-weighted imaging; ADC: apparent diffusion coefficient; T1W1: T1-weighted imaging

Postoperatively, the patient was admitted to the surgical intensive care unit, remained intubated for one day, and was then extubated and shifted to the neurosurgical ward after four days. A postoperative MRI of the brain was performed (Figures 4 and Figure 5), which showed interval improvement in the mass effect on surrounding structures, including the brainstem, left cerebellar hemisphere, third and fourth ventricles, and cisternal segments of the left facial, vestibulocochlear, and trigeminal nerves, with no hydrocephalus.

Tumor residual was noted in the cerebrospinal fluid (CSF) space medial to the temporal horn of the left lateral ventricle, with its superior margin indenting the ipsilateral occipital horn. No other definite residual was observed. The patient developed cerebellar mutism and was unable to verbalize any words, along with gait imbalance, dysmetria, and dysphagia to thin liquid fluids. These new postoperative symptoms gradually improved over two weeks. He was then discharged home and given an appointment in the outpatient clinic.

Two weeks later, the patient was seen in the clinic. He was doing well and had recovered from all postoperative transient symptoms, but the left sensorineural hearing loss remained unchanged from the preoperative state, according to him. The patient was scheduled to see an ENT physician for a complete auditory evaluation, and we will continue to follow him clinically and with serial brain imaging in the outpatient clinic.

## Discussion

Epidermoid cysts, also known as inclusion cysts, are typically composed of squamous epithelium and filled with keratin debris. They most often occur in the CPA, a location that can lead to various clinical manifestations due to compression of nearby structures, particularly cranial nerves [5,6]. The presentation of hearing loss as the sole symptom is rare. Our case is unique in this regard, as the patient exhibited no other typical signs of CPA lesions, such as vertigo, facial weakness, or neurological deficits, despite the large size and extent of the tumor and the compression and displacement evident on brain imaging [6].

This case emphasizes the importance of maintaining a high index of suspicion when evaluating patients who present with isolated unilateral sensorineural hearing loss. Considering rare pathologies early can significantly influence the outcome.

Imaging plays a crucial role in diagnosing epidermoid cysts. Typical MRI features include low signal intensity on T1-weighted images, high signal intensity on T2-weighted images, and diffusion restriction on DWI-findings that are characteristic of epidermoid cysts [6]. In our patient, MRI findings were consistent with this classic presentation, further supporting the diagnosis. The cyst encased the basilar artery, left vertebral artery, and left posterior cerebral artery, which is a common feature of epidermoid cysts due to their slow growth and tendency to displace surrounding structures without direct invasion [7]. Additionally, peripheral scattered contrast enhancement in some cases, along with midline shift on imaging, may suggest a large, slowly growing lesion with significant mass effect [8].

The differential diagnosis of CPA lesions based on imaging characteristics is broad and includes vestibular schwannomas, meningiomas, arachnoid cysts, and others. Table 1 summarizes these differential diagnoses based on typical imaging features. Understanding the subtle differences between these pathologies is essential for accurate diagnosis and appropriate management. Epidermoid cysts, in particular, may be mistaken for other lesions, but their characteristic MRI features - especially diffusion restriction on DWI and absence of enhancement - can help distinguish them from malignant tumors [9].

Surgical management remains the gold standard and mainstay of treatment for epidermoid cysts, with total excision being the most definitive approach. In cases where complete resection is not feasible due to the cyst’s adherence to critical neurovascular structures [10], as in our patient, a residual cyst may remain, and close follow-up is essential. Monitoring should continue for up to five years due to the slow growth rate of these cysts [10,11].

In our case, the patient exhibited transient cerebellar signs, likely due to postoperative changes and edema. These symptoms resolved within two weeks, and follow-up imaging showed significant improvement, including resolution of the midline shift and normalization of ventricular size [11]. Persistent hearing loss was noted, likely due to irreversible damage to the vestibulocochlear nerve from prolonged compression [11]. This underscores the importance of early intervention to prevent permanent neurological sequelae, especially when symptoms such as hearing loss are the primary presenting complaint.

Despite the rarity of epidermoid cysts, it is critical for clinicians, particularly those in ENT and Neurosurgery, to maintain a high index of suspicion when encountering unexplained hearing loss, whether isolated or accompanied by other neurological signs. Timely imaging and referral to a multidisciplinary team can lead to earlier diagnosis and more favorable outcomes, as demonstrated in this case.

## Conclusions

This case of a large epidermoid cyst presenting with isolated unilateral hearing loss highlights the rarity of such presentations, particularly in the context of a cyst of this size and extent. Despite the encasement, displacement, and kinking of multiple structures, hearing loss was the only presenting symptom. This emphasizes the importance of a thorough diagnostic evaluation. Early identification and timely intervention can help mitigate the neurological consequences of these lesions. Surgical resection is the mainstay of management, but complete excision may not always be possible, as the goal is not to induce deficits in the patient but to remove the lesion and prevent further growth and compression of neurovascular structures. However, cases with residual cyst or subtotal resection require careful monitoring, and follow-up remains crucial to prevent recurrence and manage long-term complications.
